# Unleashing the Power of Digital Voice for Early Symptom Detection for Alzheimer's Disease and Related Dementias

**DOI:** 10.1002/alz70856_101171

**Published:** 2025-12-24

**Authors:** James Glass, Liming Wang, Saurabhchand Bhati, Cody Karjadi, Meysam Ahangaran, Mahdi Khemakhem, Ian Wong, Xavier Serrano, Edward Searls, Julia Peterson, Marisa Long, Philip Joung, Vijay Sureshkumar, Rhoda Au, Lampros Kourtis, Vijaya B. Kolachalama

**Affiliations:** ^1^ Massachusetts Institute of Technology, Cambridge, MA, USA; ^2^ Framingham Heart Study, Framingham, MA, USA; ^3^ Boston University Chobanian & Avedisian School of Medicine, Boston, MA, USA; ^4^ Gates Ventures, Seattle, WA, USA

## Abstract

**Background:**

Analysis of digital voice (dVoice) is emerging as an inclusive approach to detecting the earliest preclinical symptoms of Alzheimer's disease (AD) and related dementias (ADRD) because of the widespread penetration of recording devices, such as the smartphone. Speaking is a cognitive complex task and includes concomitantly embedded neuropsychiatric related features. However, the promise of digital voice is impeded by inherent personal identifying information (PII) in the voice print and the lack of automated processing tools to extract AD/ADRD features of interest. The digital workgroup of the Global Research and Imaging Platform (GRIP) is developing open‐source tools to remove these barriers to unleash dVoice's scientific potential.

**Method:**

Leveraging >33,000 longitudinal digital voice recordings collected from the Framingham Heart Study (FHS) participants using different fidelity recording devices between 2005‐current, we have developed and tested 1) a fictitious voice conversion (FVC) method that masks the original voice print while preserving audio features, 2) a suite of automated audio, linguistic and paralinguistic feature extraction tools 3) a natural language processing (NLP) framework to splice PII from dVoice transcripts and 4) privacy protecting (PP) analysis pipeline for AI driven‐analysis.

**Result:**

We've applied the FVC method to 92 FHS recordings, ADReSS, and LibriTTS. Using version 1 of our automated feature extraction tools, we extracted acoustic, linguistic and paralinguistic features in all FHS dVoice recordings. We've applied the NLP‐PII framework to >350 manual transcriptions of FHS dVoice recordings that include marked PII. We've analyzed 128 FHS recordings, DementiaBank (Delaware), and ADReSS with the PP‐AI analytic approach. These tools have been released by GRIP in its modularly organized system, allowing users to select those that are relevant to their dVoice workflows. FVC preservation of audio features that cannot be reversed engineered did not reach sufficient levels of analysis comparability compared to dVoice recordings in their native format.

**Conclusion:**

The GRIP v1 release of dVoice processing tools are sufficiently robust to be used on recordings with varying levels of fidelity. The availability of these tools facilitates studies using voice recordings to explore their utility for measuring cognitive and behavioral symptoms of early AD/ADRD, including during the preclinical stage.

